# HIV-1 Tat-Mediated Apoptosis in Human Blood-Retinal Barrier-Associated Cells

**DOI:** 10.1371/journal.pone.0095420

**Published:** 2014-04-16

**Authors:** Xin Che, Fanglin He, Yuan Deng, Shiqiong Xu, Xianqun Fan, Ping Gu, Zhiliang Wang

**Affiliations:** Department of Ophthalmology, Ninth People’s Hospital, Shanghai Jiao Tong University, Shanghai, China; Medical University of South Carolina, United States of America

## Abstract

HIV-1-associated ocular complications, such as microvasculopathies, can lead to the loss of vision in HIV-1-infected patients. Even in patients under highly active antiretroviral therapy, ocular lesions are unavoidable. Ocular complications have been demonstrated to be closely related to the breakdown of the blood-retinal-barrier (BRB); however, the underlying mechanism is not clear. The data from this study indicated that the HIV-1 Tat protein induced the apoptosis of human retinal microvascular endothelial cells (HRMECs) and retinal pigmen epithelium (RPE) cells, which compose the inner BRB and the outer BRB, respectively. In addition, this study found that the activation of N-methyl-D-aspartate receptors (NMDARs) was involved in the apoptosis of RPE cells, but it caused no changes in HRMECs. Furthermore, both cell types exhibited enhanced expression of Bak, Bax and Cytochrome c. The inhibition of Tat activity protected against the apoptosis induced by NMDAR activation and prevented the dysregulation of Bak, Bax and Cytochrome c, revealing an important role for the mitochondrial pathway in HIV-1 Tat-induced apoptosis. Together, these findings suggest a possible mechanism and may identify a potential therapeutic strategy for HIV-1-associated ocular complications.

## Introduction

Despite the advent of highly active antiretroviral therapy (HAART), ocular lesions still occur as complications in HIV patients. Cytomegalovirus retinitis (CMVR) is the most common HIV ocular complication, and microvasculopathy, which is called HIV retinopathy in patients without ocular opportunistic infections, has the second-highest prevalence (9.4%) [1], which could result in a high incidence of blindness within the HIV-infected population [2], [3]. A detectable HIV-1 viral load has been found in tears, even in patients under long-term HAART who have an undetectable plasma viral load [4]. This finding prompts the question: how does HIV-1 induce the break-down of the blood-retinal-barrier (BRB) and invade ocular tissues?

The BRB is composed of human retinal microvascular endothelial cells (HRMECs) and retinal pigment epithelium (RPE), which provide a dynamic barrier that regulate the bidirectional movement of signals responsible for the control of eye homeostasis [5]–[7]. The posterior part of the uvea, or the choroid, is one of the most highly vascularized tissues in the body; its main function is to supply oxygen and nutrients to the outer retina, primarily to the RPE [8]. The capillaries in the retina are the continuous type and constitute the BRB. However, the capillaries of the choroid are fenestrated and contain especially large pores, which are highly permeable not only to glucose but also to low-molecular-weight substances, thereby facilitating transport across the RPE to the retina [9]. Therefore, the RPE acts as an important outer barrier to prevent the movement of pathogenic microorganisms or substances from the blood into the eye. We hypothesized that HIV-1 moves across the RPE to invade the ocular tissues, and the persistence of HIV-1 in the eye may lead to the formation of an ocular reservoir.

Retinal lesions, such as microaneurysms and hemorrhages, have been linked to the disruption of the BRB [10] and can lead to neuronal and glial cell damage. Indeed, cotton wool spots are signs of ischemic infarction in the retinal nerve fiber layer [11], [12]. HRMECs are located between blood and tissues, and their dysfunction and/or injury play a pivotal role in the development of retinopathy. Numerous studies have identified the profound effects of HIV-1 on endothelial cells (ECs), which result in several vascular disorders characterized by an evident activation and perturbation of ECs. These disorders include vasculitis in several organs [13]–[15], which leads to a high incidence of stroke [16], retinal pathology [12] and cardiovascular diseases [17].

Tat, the transactivator protein of HIV-1, plays critical and complex roles in both the HIV-1 replication cycle and the pathogenesis of HIV-1 infection. However, the effects of HIV-1 Tat on HRMECs and RPE cells have not been well elucidated. In this study, we found that the Tat-induced apoptosis of HRMECs and RPE may regulated by the mitochondrial pathway. The present study may point to novel mechanisms responsible for the HIV-induced apoptosis of HRMECs and RPE cells, which is involved in the breakdown of the BRB.

## Materials and Methods

### Cell Culture and Treatment

Human retinal microvascular endothelial cells (HRMECs, ACBRI 181) were purchased from Cell Systems Corporation (Kirkland, WA) [18] and were cultured in DMEM/F12 supplemented with 15% fetal bovine serum and 30 mg/ml endothelial cell growth factor (Invitrogen, California, USA). Human retinal pigment epithelial cells (ARPE-19) were purchased from the American Type Culture Collection (ATCC, Manassas, VA) [19], and D407 cells were obtained from the Central Laboratory of Central South University Xiangy. Both cell lines were cultured in DMEM/F12 medium (Invitrogen, Carlsbad, CA) supplemented with 10% fetal bovine serum (Invitrogen). The cells were incubated at 37°C in a humidified atmosphere of 5% CO_2_. For experiments, the cells were seeded onto 12- or 6-well plates, depending on the specific experimental requirements.

HIV-1 Tat protein is released from HIV-infected cells and is found circulating in the blood of HIV-1-infected patients [20]. Therefore, cells were treated with Tat at a concentration of 0, 200, 400 or 600 ng/ml, respectively [21]. The HIV-1 Tat protein (PROSPEC, Israel) is a non-glycosylated polypeptide chain containing 86 amino acids.

### Immunocytochemistry

Cells were grown in 12-well plates on glass coverslips (VWR, West Chester, PA) coated with laminin (Sigma-Aldrich, Saint Louis, MO). The cells were fixed with 4% paraformaldehyde (Sigma-Aldrich), permeabilized with 0.3% Triton X-100 (Sigma-Aldrich) in PBS, and blocked with 10% normal goat serum (Invitrogen). The cells were then subjected to immunofluorescence staining using a rabbit polyclonal anti-Tat antibody (Abcam, Cambridge, Massachusetts, 1∶500), and Annexin V-FITC (Invitrogen). Fluorescently labeled secondary antibodies (goat anti-mouse/rabbit Alexa Fluor 488, BD, 1∶800) were used. After washes, the cell nuclei were counterstained with propidium iodide (PI; Invitrogen). Negative control samples were processed in parallel. Immunoreactive cells were visualized and the images were recorded using a fluorescence microscope (Olympus BX51, Japan).

### Flow Cytometry

Apoptotic cells were identified via a flow cytometric analysis using an Annexin V-FITC kit (Invitrogen). The samples were washed twice with ice-cold PBS, stained with Annexin-V and PI for 20 minutes in the dark, diluted in 400 µl of binding buffer and assayed using flow cytometry according to the manufacturer’s instructions.

### Total RNA Isolation and Quality Controls

Total RNA was extracted from the cultured cells using Trizol (Invitrogen) according to the manufacturer’s instructions. The samples were digested with DNase I to avoid genomic DNA contamination. The concentration and purity of the total RNA were determined spectrophotometrically at OD260 nm and OD280 nm. Samples with OD260/280 nm ratios between 1.9 and 2.1 were used for cDNA synthesis.

### Reverse Transcription and quantitative Polymerase Chain Reaction (qPCR)

One microgram of total RNA was reverse transcribed into cDNA in a final reaction volume of 10 µl using the PrimeScript™ RT reagent kit (Perfect Real Time, TaKaRa, Dalian, China) or the miRcute miRNA first-strand cDNA synthesis kit (TIANGEN Biotech Co., Ltd. Beijing, China). The resulting cDNAs were diluted 20-fold in nuclease-free water (Invitrogen) and were used as templates for qPCR. qPCR was performed in 20 µl reactions containing 10 µl of 2× SYBR Premix EX Taq™ (TaKaRa) or 10 µl of 2× miRcute miRNA premix (TIANGEN), 2 µl of cDNA, and 300 nM of gene-specific primers (Table 1). qPCR was performed using the 7500 Real-Time PCR Detection System (Applied Biosystems, Foster, CA). The efficiency of the reaction was measured with primers using serial dilutions of the cDNA (1∶1, 1∶5, 1∶25, 1∶125, 1∶625 and 1∶3,125). Each sample was tested in triplicate. The relative mRNA expression was analyzed using the Pfaffl method [22]. The data were normalized to the expression of *GAPDH* and are expressed as fold changes relative to untreated controls.

**Table 1:** Primers used for quantitative RT-PCR.

Genes	Accessionno.	Forward(5′-3′)	Reverse(5′-3′)	Annealingtemperature (°C)	Product size(base pairs)
NMDAR	NM_001185091	ctaccgcatacccgtgctg	gcatcatctcaaaccacacgc	60	128
Bcl-2	NM_000633	atgtgtgtggagagcgtcaa	gggccgtacagttccacaaa	60	143
Bax	NM_138763	cccgagaggtctttttccgag	ccagcccatgatggttctgat	60	155
Bak	NM_001188	atggtcaccttacctctgcaa	tcatagcgtcggttgatgtcg	60	97
Cytochrome c	NM_018947	gagcgggagtgttcgttgt	cttccgcccaaagagaccat	60	165
GAPDH	NM_001256799	ctgggctacactgagcacc	aagtggtcgttgagggcaatg	60	101

### Western Blotting Analysis

The cells were harvested at the indicated time points. Total protein was extracted and its concentration was determined using a BCA Kit (Pierce, Rockford, IL) according to the manufacturer’s protocol. Proteins were separated using sodium dodecyl sulfate-polyacrylamide gel electrophoresis (SDS-PAGE) and were then transferred to polyvinylidene fluoride (PVDF) membranes (Millipore, Bedford, MA). After being blocked with 5% nonfat milk, the membranes were incubated with rabbit polyclonal anti-NMDAR antibody (Abcam, Cambridge, Massachusetts, 1∶500), anti-Bak antibody (Abcam, 1∶1000), anti-Bax antibody (Abcam, 1∶1000), anti-cytochrome c antibody (Proteintech, Chicago, Illinois, 1∶500), or rabbit anti-β-actin antibody (Sigma, 1∶0000) at 37°C for 2 h, followed by incubation with horseradish peroxidase (HRP)-conjugated secondary antibodies (1∶5000, Sigma). Protein expression was visualized using Odyssey V 3.0 image scanning software (LI-COR, Lincoln, NE). Semi-quantification of the protein concentrations was accomplished on the basis of three independently performed experiments. The densitometric intensities of the protein bands were quantified using Bandscan 5.0 software, and the values were normalized against β-actin for each sample.

### Knockdown of N-methyl-D-aspartate Receptor (NMDAR)

Cells were plated onto 6-well plates. Complexes of siRNA duplexes and Lipofectamine 2000 (Invitrogen) were prepared as follows: 5 µl of 20 µM siRNA was diluted in 500 µl of Opti-MEM I medium (Invitrogen), mixed with 5 µl of Lipofectamine 2000 and incubated for 15 min. The complexes were added to each well. The cells were incubated at 37°C in a 5% CO_2_ humidified incubator for 6 h. The medium was then replaced with DMEM/F12, and the cells were incubated for an additional 18 h for further analysis. The targeted siRNA-NMDAR sequences were 5′-CAC CGG ACG GGT AGA ATT CAA-3′ and 5′-ACG CAT GTC TAT ATA TTC TGA-3′. The negative control siRNA sequences were 5′-UUU UCC GAA CGU GUC ACG UTT-3′ and 5′-ACG UGA CAC GUU CGG AGA ATT-3′ (GenePharma, China).

### Statistical Analyses

Each experiment was performed at least three times unless otherwise specified. The statistical data are expressed as the means ± the standard deviation (SD). The statistical analyses were performed using Student’s t-test, and statistical significance was defined as p≤0.05.

## Results

### Apoptosis of HRMECs Induced by HIV-1 Tat

**Figure 1:**
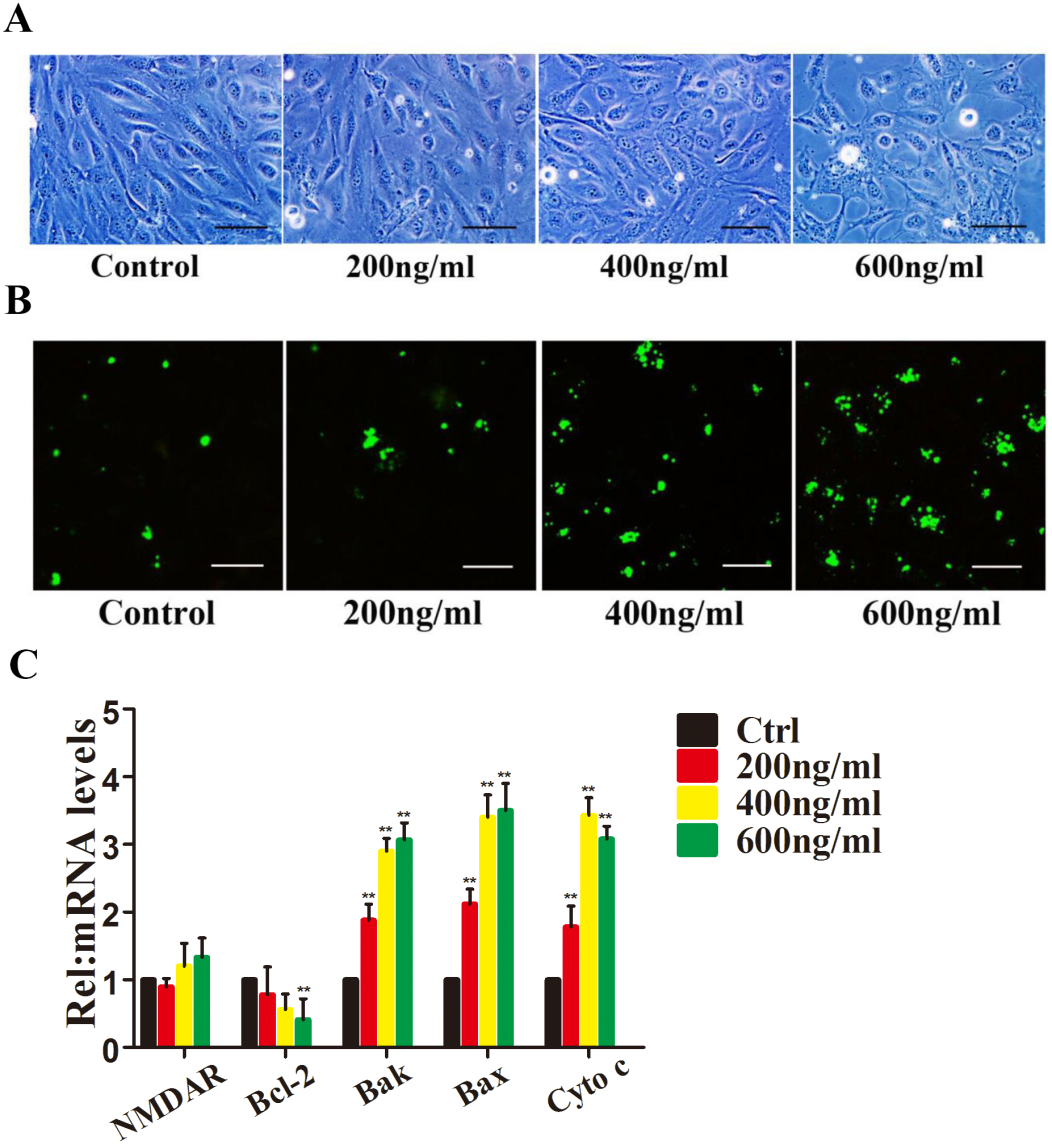
HIV-1 Tat induces apoptosis of HRMECs. HRMECs were incubated with 0, 200, 400, or 600/ml Tat for 48 hours. Representative phase-contrast microscopic images are shown, indicating the loss and swelling of HRMECs at different degrees (**A**). Immunofluorescence micrographs showing apoptosis using Annexin V-FITC staining (green). Scale bar: 50 µm (**B**). Quantitative analysis of Bcl-2, Bax, Bak and Cytochrome c expression using qPCR (*, P<0.05 vs. control) after HRMECs were treated with Tat at different concentrations for 48 h.

To investigate the effects of Tat on HRMEC apoptosis *in vitro*, apoptotic cells were identified using representative phase-contrast microscopy, which showed the loss and swelling of HRMECs at different levels (P<0.05 vs. control) (Fig. 1A). Immunocytochemistry revealed that 2±1.77%, 5±2.93%, 31±4.25%, and 40±3.06% of the cells were apoptotic after treatment with different concentrations of Tat, respectively (p≤0.05) (Fig. 1B).

The Bcl-2 family of proteins is one of the best-characterized regulators of apoptosis; it includes antiapoptotic members, such as Bcl-2, and the pro-apoptotic members Bak, Bax and cytochrome c [23], [24]. The proteins of this family mainly contribute to the mitochondrial apoptosis pathway. We hypothesized that the mitochondrial pathway may be associated with Tat-mediated apoptosis, so we next examined changes in Bcl-2, Bak, Bax and cytochrome c. After a 48 h interaction with Tat, the Bcl-2 mRNA level decreased in a dose-dependent manner, and HRMECs treated with 200 or 400 ng/ml Tat showed minimal changes (19.2 and 26.4%, respectively), likely due to the low level of Tat (Fig. 1C). In contrast, the mRNA levels of Bak, Bax and cytochrome c increased dramatically.

### Neutralizing Tat Attenuates Tat-induced HRMEC Apoptosis

**Figure 2:**
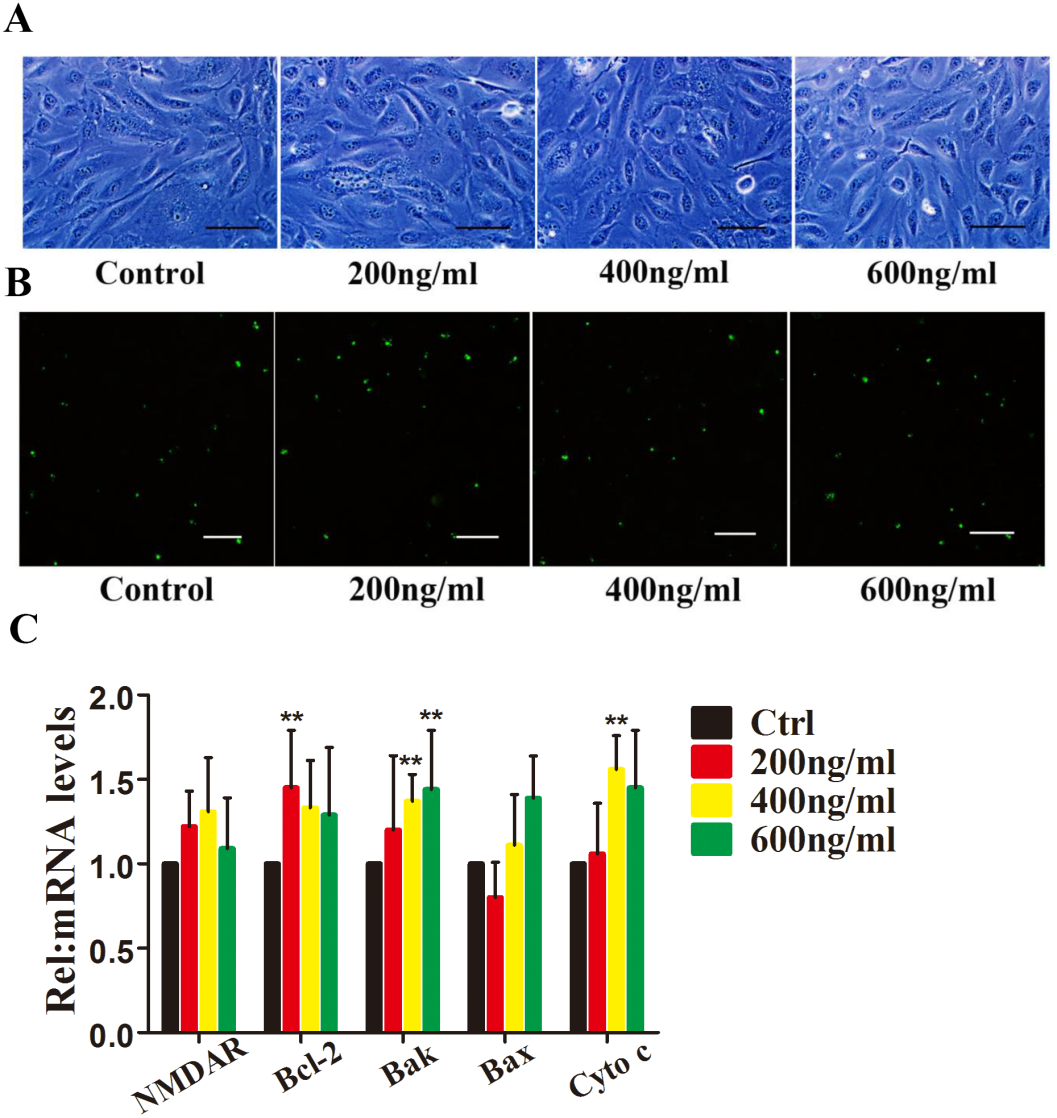
Neutralizing Tat attenuates Tat-induced HRMEC apoptosis. An anti-Tat antibody was added to HRMECs and incubated for 24 hours. The cells were then incubated with 0, 200, 400, or 600 ng/ml Tat for 48 hours. Representative phase-contrast microscopic images show the morphology of the cells (**A**). Immunofluorescence micrographs showing apoptosis using Annexin V-FITC staining (green). Scale bar: 50 µm (**B**). The levels of Bcl-2, Bax, Bak and Cytochrome c expression were quantitatively analyzed using qPCR (*, P<0.05 vs. control).

To further determine the relationship between Tat and HRMEC apoptosis, an anti-Tat antibody was used to neutralize the function of the Tat protein. HRMECs were cultured with a control IgG and an anti-Tat antibody for 24 h, followed by incubation with the Tat protein for 48 h. Phase-contrast microscopy revealed normal cell morphology (Fig. 2A). Furthermore, immunocytochemistry indicated little apoptosis (5.9%), even at 600 ng/ml Tat (Fig. 2B).

To confirm the dysfunction of the Bcl members induced by Tat, the expression levels of Bcl family proteins were measured. After treatment with the Tat antibody for 24 h, a Tat peptide was added to the HRMECs. A qPCR analysis showed that the expression of Bcl-2 was not significantly altered. Similarly, no upregulation of Bak, Bax or Cytochrome c was detected (Fig. 2C). These data confirm the results shown in Fig. 1C, which indicate that the Bcl-2, Bak, and Bax proteins participate in the Tat-induced apoptosis of HRMECs.

### Localization of HIV-1 Tat in RPE

To determine whether Tat can invade cells, D407 cells were cultured with 200 ng/ml Tat for 48 h. A confocal microscopic analysis (Fig. S1) of cells stained with the anti-Tat antibody showed that Tat localized to both the cell nucleus and the cytoplasm.

### Apoptosis of ARPE-19 Induced by HIV-1 Tat

**Figure 3:**
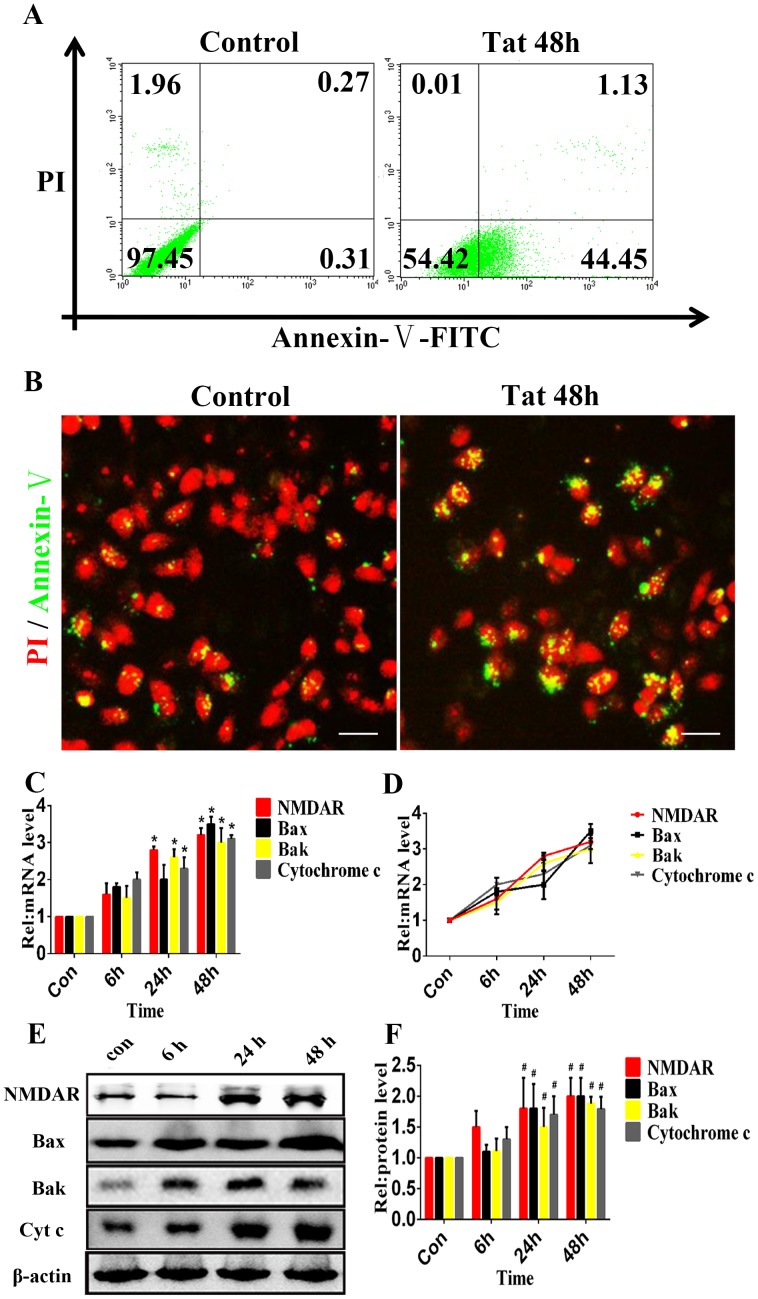
HIV-1 Tat induces apoptosis in RPE cells. ARPE-19 cells were incubated with or without 200 ng/ml Tat for 48 hours. Representative flow cytometry images are shown, which indicate the percentage of cells in early apoptosis using Annexin V-FITC staining (**A**). Immunofluorescence micrographs showing apoptosis using Annexin V-FITC staining (green); the nuclei were counterstained with PI (red). Scale bar: 50 µm (**B**). Both the flow cytometric analysis and immunofluorescence showed that Tat induced the apoptosis of RPE cells. The expression levels of NMDAR, Bax, Bak and Cytochrome c were quantitatively analyzed using qPCR (*, P<0.05 vs. control) (**C**) (**D**) and Western blotting (**E**) after ARPE-19 cells were treated with Tat for 0, 6, 24 or 48 h. The corresponding graphs show the quantification of 3 independent experiments performed in duplicate; the data were normalized to cells without Tat treatment (**#**, P<0.05 vs. control) (**F**).

The flow cytometric analysis revealed apoptosis in 45.58±3.75% of ARPE-19 cells compared with 0.58±0.98% of the control cells (p≤0.05) (Fig. 3A). Immunocytochemistry showed that in the presence of Tat, 49±2.34% of cells were apoptotic, whereas in the absence of Tat, 0.5±0.12% of cells were apoptotic (p≤0.05) (Fig. 3B). These data provide evidence that HIV-1 Tat can lead to ARPE-19 apoptosis. In addition, D407 cells were used to investigate the effect of Tat on cell apoptosis. The data from phase-contrast microscopy revealed the dose-dependent loss and swelling of D407 cells (Fig. S2-A). Furthermore, the flow cytometric analysis showed that the apoptosis of D407 cells was induced by HIV-1 Tat in a dose-dependent manner (Fig. S2-B). It has been reported that in human primary neurons, Tat-induced apoptosis is dependent on NMDAR activity [25]. NMDARs are present on RPE membranes in culture [26], implying that the apoptosis of RPE cells may be dependent on NMDAR activity. After Tat treatment for 0, 6, 24 or 48 h, quantitative analyses using qPCR (Fig. 3C and 3D) and western blotting (Fig. 3E and 3F) demonstrated the time-dependent elevation of NMDAR, Bak, Bax and Cytochrome c expression. A three-fold upregulation of NMDAR expression was evident as early as 24 h, and the upregulation of Bak, Bax and Cytochrome c corresponded with the degree of NMDAR upregulation. These data suggest that HIV-1 Tat can upregulate the expression of NMDAR in ARPE-19 and D407 cells, and the elevated expression of Bak and Bax may be responsible for the apoptosis of RPE cells (Fig. S2 and S3). However, we found that Tat induced minimal changes in the expression of NMDARs on HRMECs (Fig. 1C and 2C).

### Blocking Tat Protected against Tat-induced RPE Apoptosis

**Figure 4:**
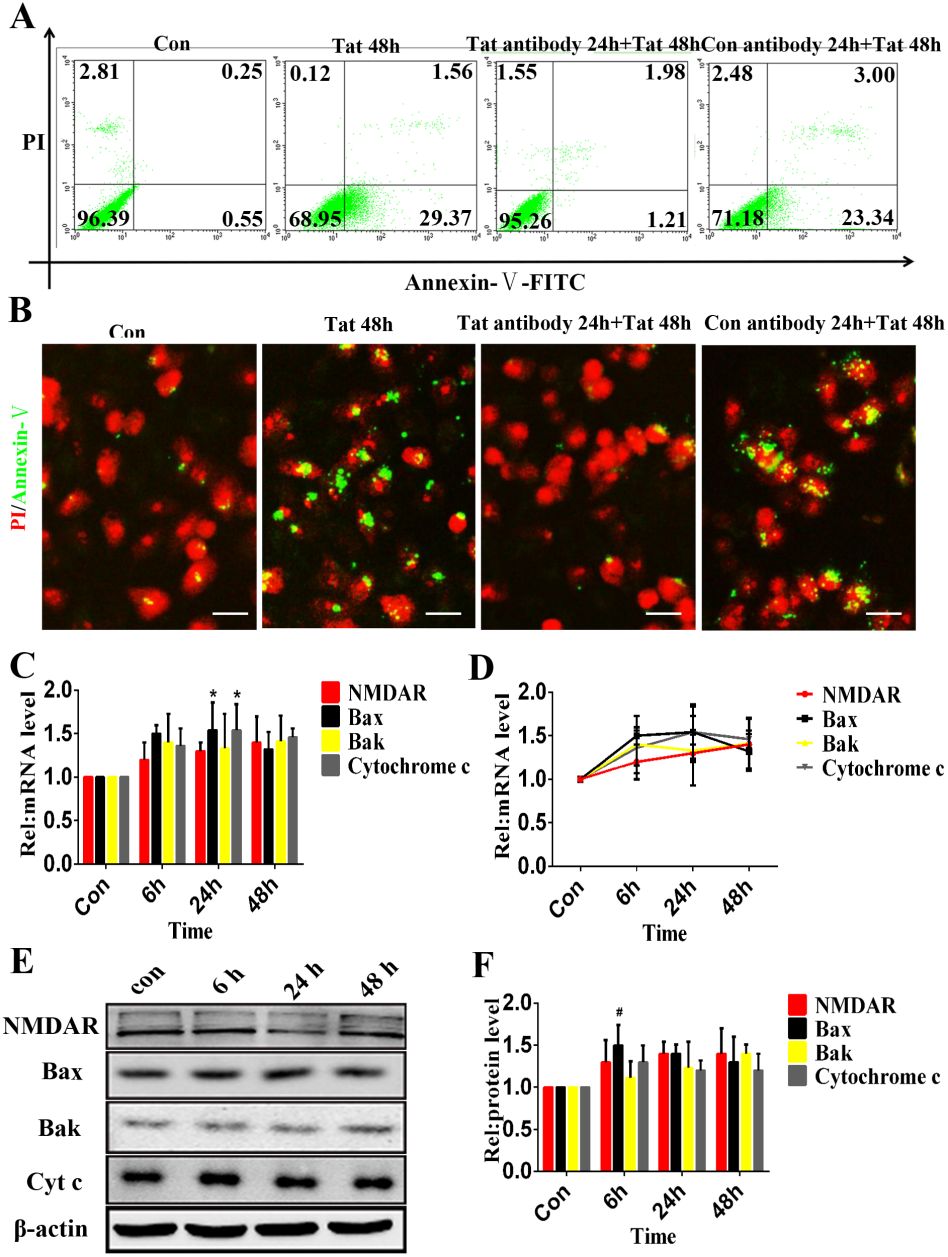
Inactivation of Tat protected against Tat-induced RPE apoptosis. An anti-Tat antibody was added to the ARPE-19 and incubated for 24 hours, and the cells were then incubated with or without 200 ng/ml Tat for 48 hours. Representative flow cytometry images showing the percentage of cells in early apoptosis using Annexin V-FITC staining (**A**). Immunofluorescence micrographs showing apoptosis using Annexin V-FITC staining (green) with nuclei counterstained with PI (red). Scale bar: 50 µm (**B**). Both of these analyses showed that the Tat antibody protected ARPE-19 cells from the apoptosis induced by Tat. After incubation with the Tat antibody for 24 h, the ARPE-19 cells were cultured with Tat for 0, 6, 24 or 48 h. The expression levels of NMDAR, Bax, Bak and Cytochrome c were quantitatively analyzed using qPCR (*, P<0.05 vs. control) (**C**) (**D**) and Western blotting (**E**). The corresponding graphs show the quantification of 3 independent experiments performed in duplicate; the data were normalized to cells without Tat treatment (**#**, P<0.05 vs. control) (**F**).

As described above, ARPE-19 and D407 cells were cultured with an anti-Tat antibody and a control IgG for 24 h and were then incubated with the HIV Tat protein for 48 h. A flow cytometric analysis revealed that the Tat antibody treatment reduced apoptosis by 27.74±0.47% (Fig. 4A). Immunocytochemistry showed similar apoptosis levels, which suggested that the Tat antibody attenuated apoptosis by 29.73±2.58% (Fig. 4B).

The ARPE-19 and D407 cells were pretreated with the Tat antibody for 24 hours and were then treated with Tat protein for 0, 6, 24 or 48 h. The expression levels of NMDAR, Bak, Bax and Cytochrome c were analyzed using qPCR (Fig. 4C and 4D) and western blotting (Fig. 4E and 4F). Our data showed that the expression of NMDAR, Bak, Bax and Cytochrome c did not significantly change over time, with a maximum increase of 1.5-fold compared with the control (1.5 vs. 1.0, p>0.05), suggesting that the effects of HIV-1 Tat treatment were specifically due to the Tat protein; the anti-Tat antibody evidently neutralized the effects of Tat on ARPE-19 and D407 cells (Fig. S4).

### Silencing of NMDAR Abolished Tat-induced RPE Apoptosis

**Figure 5:**
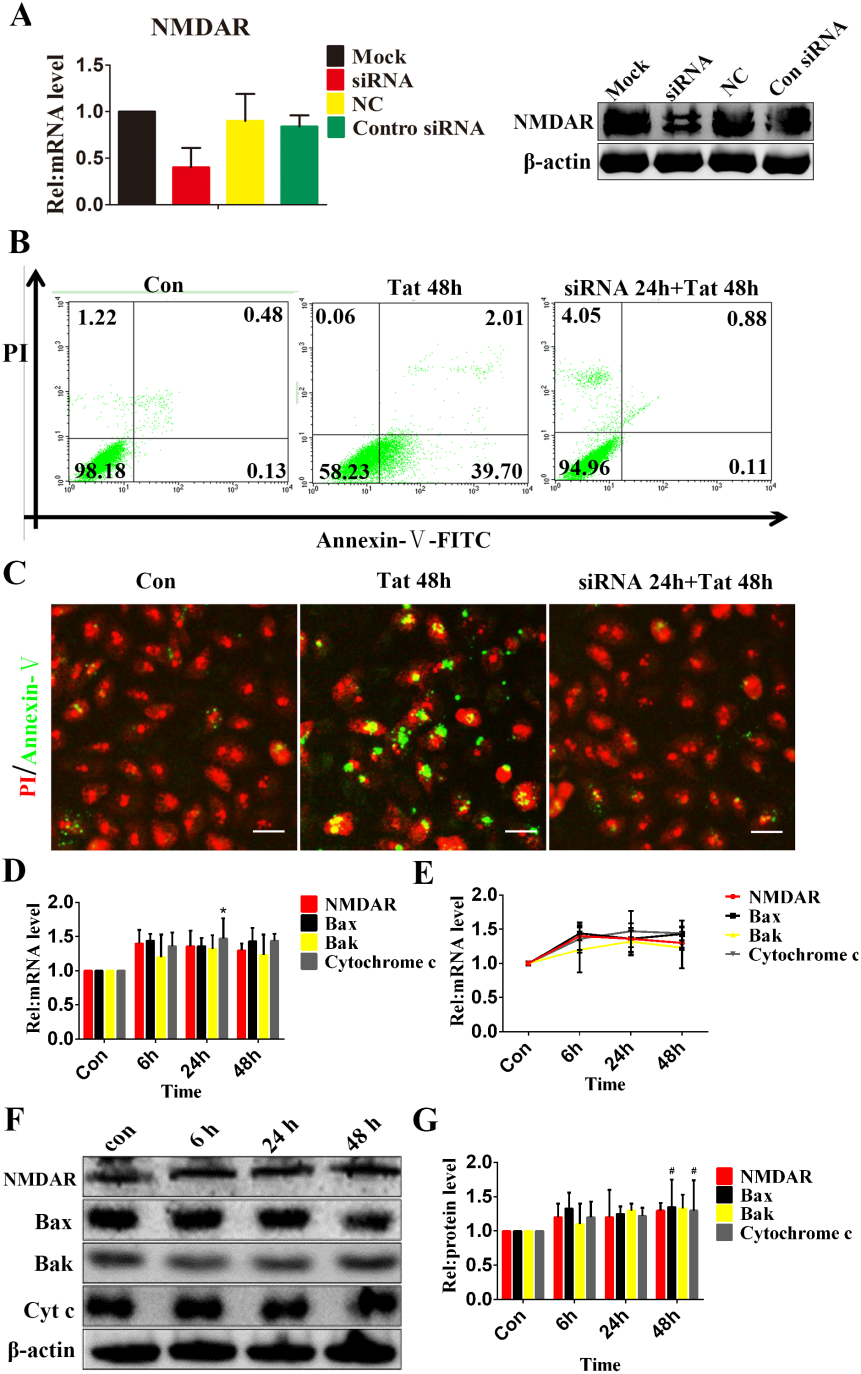
Silencing of NMDARs inhibits Tat-induced RPE apoptosis. ARPE-19 cells were transfected with NMDAR1-specific small interfering RNA for 24 hours, and the degree of NMDAR1 silencing was estimated using qPCR and Western blotting (**A**). Representative flow cytometry images show early apoptotic cells in which NMDAR was knocked down for 24 h, Tat was added, and incubation continued for an additional 48 h. Apoptotic cells were visualized using Annexin V-FITC staining (**B**). Immunofluorescence micrographs showing apoptotic cells using Annexin V-FITC staining (green); the nuclei were counterstained with PI (red). Scale bar: 50 µm. Both of these analyses showed that the knockdown of NMDAR could protect RPE cells from the apoptosis induced by Tat (**C**). After the silencing of NMDAR1 for 24 h, ARPE-19 cells were cultured with Tat for 0, 6, 24 or 48 h. The expression levels of NMDAR1, Bax, Bak and Cytochrome c were quantitated using qPCR (*, P<0.05 vs. control) (**D**) (**E**) and Western blotting (**F**). The corresponding graphs show the quantification of 3 independent experiments performed in duplicate; the data were normalized to cells without Tat treatment (**#**, P<0.05 vs. control) (**G**).

To determine whether NMDARs specifically mediate RPE apoptosis, NMDARs were silenced using siRNA. Three distinct NMDAR subunits (NMDAR 1, NMDAR 2, and NMDAR 3) have been identified, each of which shows different activity [27]. Among these subunits, only NMDAR1 is required for NMDAR function; therefore, impairing its expression necessarily limits NMDAR function. Using siRNA corresponding to NMDAR1, the expression of NMDAR1 was reduced by ∼60% compared with mock-transfected control cultures (Fig. 5A). The decreased expression of NMDAR1 effectively protected against Tat-induced ARPE-19 apoptosis, which was reduced by 39.71±0.25% compared with the group treated with Tat alone (Fig. 5B). Additionally, immunocytochemistry showed that the decreased expression of NMDAR1 attenuated apoptosis by 40.3±2.78% (Fig. 5C).

Next, NMDAR was silenced for 24 h, and the expression levels of NMDAR, Bak, Bax and Cytochrome c were evaluated after cells were incubated with Tat for 0, 6, 24 or 48 h. Quantitative analyses using qPCR (Fig. 5D and 5E) and western blotting (Fig. 5F and 5G) demonstrated the absence of significant changes over time (Fig. 4 and Fig. S5).

## Discussion

HIV-associated damage is thought to be due to an indirect mechanism mediated by either virally infected or uninfected cells. Candidate toxins that produce HIV-associated damage include cytokines, glutamate, and virally encoded proteins, such as the HIV transactivator protein, Tat [28]. HIV-1 Tat plays critical and complex roles in both HIV-1 replication and the pathogenesis of HIV-1 infection by modulating the expression of several cellular genes and triggering the activation of certain signal transduction pathways and transcription factors [29]. The full-length HIV-1 Tat protein contains 101 amino acids, can be secreted from infected cells and is found at detectable levels in serum [20], [30], supporting the hypothesis of its role as a progression factor in the progression of AIDS. In this study, we found that the HIV-1 Tat protein could induce the apoptosis of HRMECs and RPE cells *in vitro*, and the inhibition of Tat abrogated this Tat-induced apoptosis, implying a specific apoptosis effect of Tat on HRMECs and RPE cells.

The HIV reservoir refers to the persistence of HIV in cells (such as resting CD4^+^ cells [31]) or organs (such as those of the central nervous system [31]), even in the presence of an effective immune response and antiviral therapy. Since the advent of the HAART era, there have been reports of higher viral loads in intraocular fluid than in plasma, suggesting that the ocular organ may be a viral sanctuary [32]. We hypothesized that the breakdown of the BRB may be a critical contributing factor to the entry of HIV-1 into the eye, and our data showed that HIV-1 Tat induced the apoptosis of both HRMECs and RPE cells, resulting in the loss of BRB integrity. Thus, the “opening” of a passage between the blood and eye may permit HIV-infected cells and/or HIV-associated factors to invade ocular tissues.

Several studies of the brain have demonstrated a role of NMDARs/NMDA in the apoptosis of human neurons and astrocytes via the phosphorylation of NMDARs or the formation of a NMDAR-associated complex [25], [33]. In the retina, NMDARs are widely expressed in neuronal cells [34], where they are involved in the glutamate-induced apoptosis of retinal neurons. For example, long-term injection of glutamate at low concentrations induces retinal ganglion cell death and neuronal apoptosis in rats [35], [36]. The administration of MK-801/memantine, an NMDA channel blocker, prevented retinal ganglion cell death, retinal ischemia and diabetic retinopathy in an experimental rat model of glaucoma [37]. The data from the present study demonstrated that HIV-1 Tat induced the upregulation of NMDAR expression, and neutralizing Tat attenuated the elevated expression of NMDAR, suggesting a direct link between Tat and NMDAR in the RPE. Furthermore, it has been reported that HIV-1 Tat potentiated glutamate-induced excitoxicity in nerve cells and promoted brain neuronal apoptosis via NMDARs [38]. Additionally, the knockdown of NMDARs inhibited Tat-induced apoptosis and prevented the upregulation of Bak, Bax and Cytochrome c. These results indicate that NMDARs may contribute to the HIV-1 Tat-induced apoptosis of RPE cells. However, the present data indicated that Tat induced minimal changes in NMDARs on HRMECs, a finding that requires further confirmation.

HIV-1 Tat has been reported to exert adverse effects on vascular endothelial cells, such as the disruption of capillary integrity [39], [40], the induction of inflammatory responses [41], and the apoptosis of cells [42], [43], leading to the further dysfunction of cells, tissues and even organs. Retinopathy involves the impairment of retinal capillaries. The pathophysiology of retinopathy involves many factors, with the failure of the BRB being the fundamental factor. The failure of the inner BRB is caused by the loss of endothelial cell in capillaries along with the loss of pericytes [44]. Taken together, our data indicate that the observed apoptosis of HRMECs could lead to the loss and vulnerability of BRB-associated cells, which may be related to the underlying pathophysiology.

The mechanism of Tat-induced apoptosis of HRMECs has not been well elucidated. The treatment of human brain microvascular endothelial cells (HBMECs) with HIV-1 Tat has been reported to lead to Flk-1/KDR and Flt-4 receptor activation as well as the release of NO, which stimulated the apoptosis of HBMECs and resulted in the irreversible loss of BBB integrity [42]. However, in human microvascular endothelial cells of lung origin, Tat caused apoptosis via the activation of caspase-3, a potent “executioner” enzyme that functions in apoptotic signaling [43]. In the present study, HIV-1 Tat induced the apoptosis of HRMECs by a mechanism distinct from that described above. The mitochondrial pathway of apoptosis is dependent on mitochondrial outer membrane permeabilization (MOMP), which leads to the release of proteins from the mitochondrial intermembrane space into the cytosol. This release is mediated by the proapoptotic Bcl-2 family members Bak and Bax, and it is inhibited by antiapoptotic Bcl-2 members, such as Bcl-2 proteins [45]. The results of our study indicated that Tat treatment caused the downregulation of Bcl-2 and the upregulation of Bak, Bax and Cytochrome c, both of which were dose-dependent. Therefore, we hypothesized that the dysfunction of Bcl-2, Bak, Bax and Cytochrome c may contribute to Tat-mediated apoptosis. Next, we added an anti-Tat antibody to cells followed by Tat treatment, to confirm that these proapoptotic effects were mediated by Tat. The resulting data indicated that this treatment led to minimal dysregulation of Bcl-2, Bak, Bax and Cytochrome c. Taken together, all of these data demonstrate a specific role of Tat in the apoptosis of HRMECs via the mitochondrial pathway. Similarly, Tat activated the mitochondrial pathway in RPE via the elevation of Bak, Bax and Cytochrome c. However, further research into the role of Bcl-2 in the Tat-RPE interaction is needed.

All of these results suggest that HIV-1 Tat may induce the apoptosis of HRMECs and RPE cells by the mitochondrial pathway and may activate NMDARs on RPE; thus, HIV-1 Tat may play an important role in the pathogenesis of HIV-1-associated ocular complications. Understanding the detailed molecular mechanism of the apoptosis caused by HIV-HRMEC and/or HIV-RPE interactions may provide the basis for novel therapeutic strategies to prevent ocular complications in HIV-infected patients. However, further investigations are needed to elucidate the mechanism through which Tat induces the apoptosis of HRMECs and RPE cells.

## Supporting Information

Figure S1 **HIV-1 Tat localization in RPE cells.** Confocal micrographs from D407 cells cultured with 200 ng/ml Tat for 48 hours. D407 were stained with anti-Tat antibody (green puncta). Tat is distributed in both the cytoplasm and nucleus of D407 cells. Scale bars: 100 µm.
(TIF)

Figure S2 **HIV-1 Tat induced apoptosis of RPE.** D407 were growed in the six-well plates, the cells were cultured with 0, 200, 400 and 600 ng/ml Tat for 48 hours, respectively. Representative phase contrast microscopy showed the loss and swelling of RPE at different levels (P<0.05 vs. control) **(A)**. Flow cytometry was used to determine the apoptosis, with the different concentration of Tat, the rate of apoptosis cells was significantly different (P<0.05 vs. control) **(B)**.
(TIF)

Figure S3 **HIV-1 Tat caused the upregulation of NMDAR, Bak, Bax and Cytochrome c.** The D407 were treated with 0, 200, 400 and 600 ng/ml Tat for 0, 6, 24 and 48 h, respectively, qPCR were performed to detect the changes of NMDAR, Bak, Bax and Cytochrome c at mRNA levels. The data indicated that the NMDAR, Bak, Bax and Cytochrome c were upregulated by Tat in a dose- and time-dependent manner (*, P<0.05 vs. control).
(TIF)

Figure S4 **Neutralizing Tat attenuates Tat-associated changes.** Tat antibody was added to the D407 for 24 hours, then the cells were incubated with 0, 200, 400 and 600 ng/ml Tat for 0, 6, 24 and 48 h, respectively. The expression levels of NMDAR, Bax, Bak and Cytochrome c were determined by qPCR.
(TIF)

Figure S5 **Silencing of NMDAR inhibits Tat-induced RPE changes.** D407 were transfected with NMDAR1 small interfering RNA for 24 hours, then the cells were cultured with 0, 200, 400 and 600 ng/ml Tat for 0, 6, 24 and 48 h, respectively. The expression levels of NMDAR, Bax, Bak and Cytochrome c were quantitated by qPCR.
(TIF)
