# Supplementary figures and images for: HIV-1 Tat-Mediated Apoptosis in Human Blood-Retinal Barrier-Associated Cells
Source: PLoS One. 2014 Apr 16;9(4):e95420. doi: 10.1371/journal.pone.0095420 (PMC3989329; doi:10.1371/journal.pone.0095420)

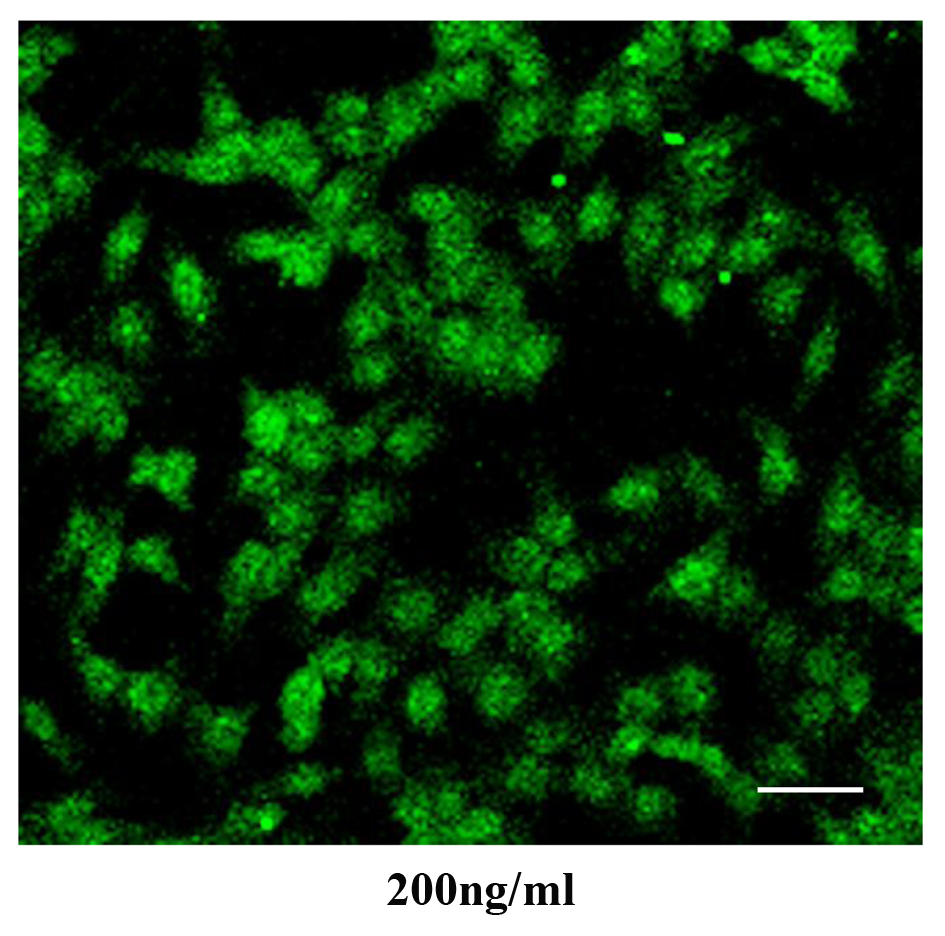

Supplement: Figure S1 — HIV-1 Tat localization in RPE cells. Confocal micrographs from D407 cells cultured with 200 ng/ml Tat for 48 hours. D407 were stained with anti-Tat antibody (green puncta). Tat is distributed in both the cytoplasm and nucleus of D407 cells. Scale bars: 100 µm. (TIF) [file pone.0095420.s001.tif]

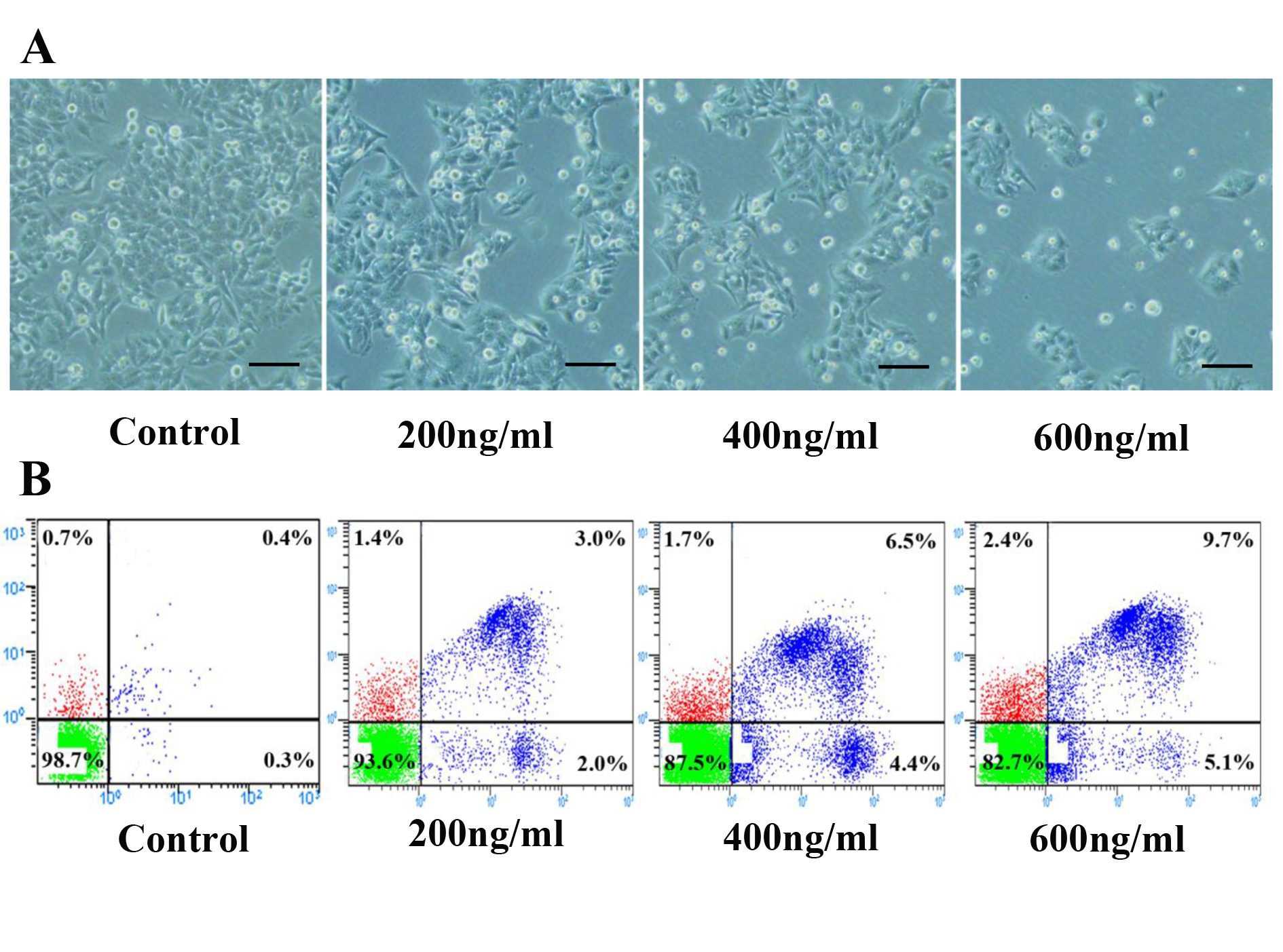

Supplement: Figure S2 — HIV-1 Tat induced apoptosis of RPE. D407 were growed in the six-well plates, the cells were cultured with 0, 200, 400 and 600 ng/ml Tat for 48 hours, respectively. Representative phase contrast microscopy showed the loss and swelling of RPE at different levels (P<0.05 vs. control) (A). Flow cytometry was used to determine the apoptosis, with the different concentration of Tat, the rate of apoptosis cells was significantly different (P<0.05 vs. control) (B). (TIF) [file pone.0095420.s002.tif]

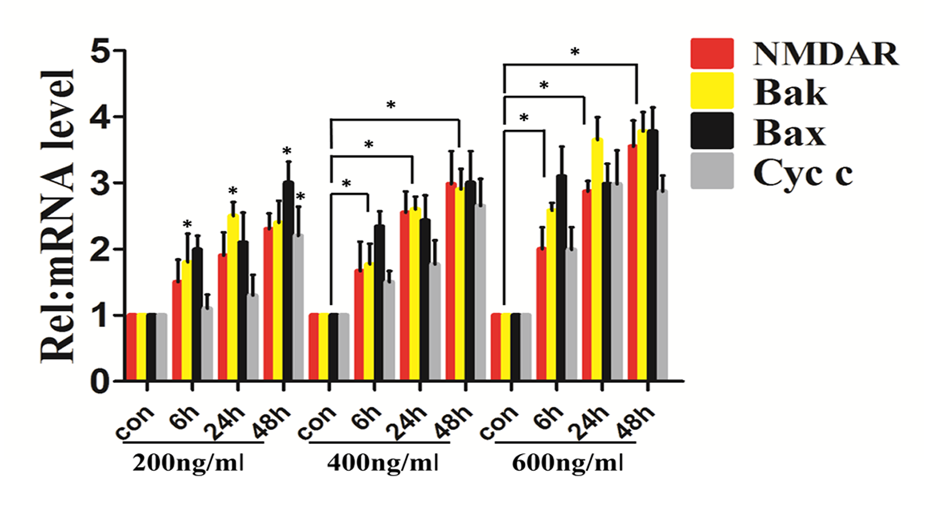

Supplement: Figure S3 — HIV-1 Tat caused the upregulation of NMDAR, Bak, Bax and Cytochrome c. The D407 were treated with 0, 200, 400 and 600 ng/ml Tat for 0, 6, 24 and 48 h, respectively, qPCR were performed to detect the changes of NMDAR, Bak, Bax and Cytochrome c at mRNA levels. The data indicated that the NMDAR, Bak, Bax and Cytochrome c were upregulated by Tat in a dose- and time-dependent manner (*, P<0.05 vs. control). (TIF) [file pone.0095420.s003.tif]

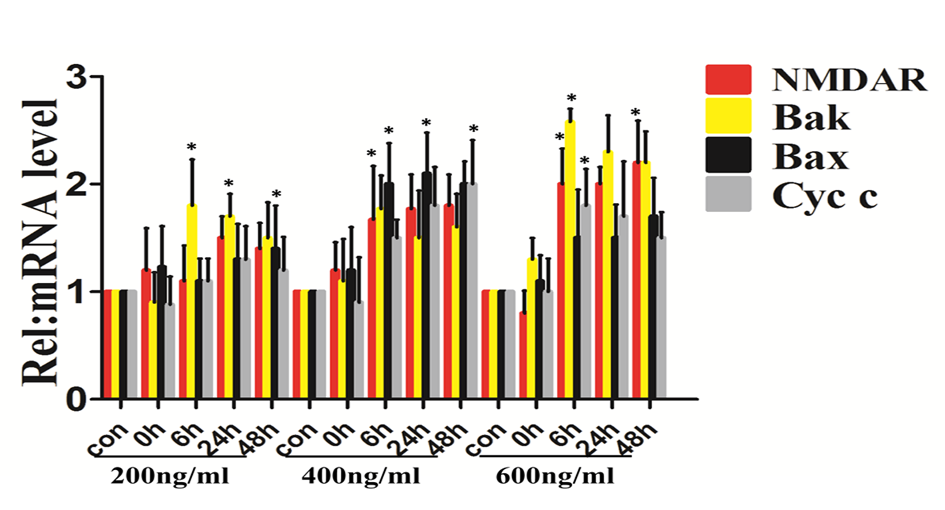

Supplement: Figure S4 — Neutralizing Tat attenuates Tat-associated changes. Tat antibody was added to the D407 for 24 hours, then the cells were incubated with 0, 200, 400 and 600 ng/ml Tat for 0, 6, 24 and 48 h, respectively. The expression levels of NMDAR, Bax, Bak and Cytochrome c were determined by qPCR. (TIF) [file pone.0095420.s004.tif]

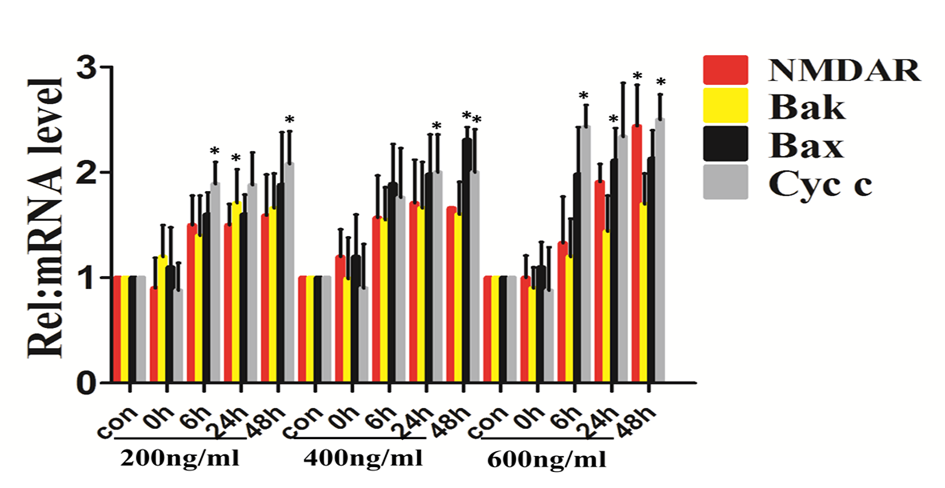

Supplement: Figure S5 — Silencing of NMDAR inhibits Tat-induced RPE changes. D407 were transfected with NMDAR1 small interfering RNA for 24 hours, then the cells were cultured with 0, 200, 400 and 600 ng/ml Tat for 0, 6, 24 and 48 h, respectively. The expression levels of NMDAR, Bax, Bak and Cytochrome c were quantitated by qPCR. (TIF) [file pone.0095420.s005.tif]
